# Extensive bilateral intracranial calcifications and seizure in iatrogenic hypoparathyroidism: A case report

**DOI:** 10.1002/ccr3.5076

**Published:** 2021-11-12

**Authors:** Parikshit Chapagain, Shambhu Khanal, Rajeev Ojha, Niraj Gautam, Matina Sayami, Roshan Bhandari

**Affiliations:** ^1^ Department of Internal Medicine Tribhuvan University Teaching Hospital Kathmandu Nepal; ^2^ Department of Neurology Tribhuvan University Teaching Hospital Kathmandu Nepal; ^3^ Department of Endocrinology Tribhuvan University Teaching Hospital Kathmandu Nepal

**Keywords:** basal ganglia calcification, hypocalcemia, hypoparathyroidism, seizure, thyroidectomy

## Abstract

Postoperative permanent hypoparathyroidism can exhibit extensive intracranial calcifications involving basal ganglia, thalamus, cerebellum, and cerebral cortex.

## Graphical abstract

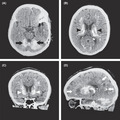

## 1 INTRODUCTION

Calcification beyond basal ganglia is rare in postoperative permanent hypoparathyroidism. We report intracranial calcifications involving basal ganglia, thalamus, cerebellum, and cerebral cortex in a 56‐year‐old Nepalese female patient with postoperative hypoparathyroidism for 20 years. She presented with tetany, seizure, and severe hypocalcemia and improved after calcium, vitamin D, and magnesium supplementation.

Postoperative permanent hypoparathyroidism is characterized by hypocalcemia, hyperphosphatemia, and low to inappropriately normal parathyroid hormone levels, which persist more than six months after surgery.^1^ It occurs in 0.3% to 5.1% of individuals who undergo thyroid surgery for thyroid cancer.^2^, ^3^, ^4^ Severe hypocalcemia can present with carpopedal spasms, tetany, seizures, and cardiac dysrhythmias. Longstanding postoperative hypoparathyroidism is characterized by intracranial calcifications of basal ganglia and cerebellum; calcifications beyond these areas are rare.^1^, ^5^ We report the case of a 56‐year‐old lady who presented with carpopedal spasms, tetany, generalized tonic‐clonic seizure, and extensive bilateral brain calcifications due to postoperative permanent hypoparathyroidism.

## 2 CASE REPORT

A normotensive, nondiabetic 56‐year‐old lady, who had undergone near‐total thyroidectomy for papillary carcinoma of thyroid 20 years ago, presented in the emergency department with history of spasms in her hands and calves, followed by sustained contraction of hand muscles. Then, she had an episode of tonic‐clonic generalized abnormal body movements with uprolling of eyes and frothing from mouth, which lasted about 30–35 seconds; it was followed by a brief period of confusion and amnesia. Twenty years ago, she had undergone near‐total thyroidectomy after a solitary thyroid nodule revealed features suggestive of papillary carcinoma of the thyroid, later confirmed by histopathological analysis of the resected mass. There was no lymphatic invasion, and she did not receive postoperative radioactive iodine therapy. Following thyroidectomy, she had been taking calcium, vitamin D, and supplemental thyroid hormone until five months ago when she stopped taking calcium and vitamin D but continued taking thyroid hormone supplement. Four months ago, she experienced episodes of calf spasms, which lasted about five minutes and were severe enough to limit walking. The episodes were empirically treated with calcium and cholecalciferol for a short duration without checking calcium levels. She had undergone bilateral cataract surgery eight years ago. She had no history of fever, headache, vomiting, palpitation, or chest pain.

On examination, the patient had positive Trousseau's sign on tourniquet test, which manifested as carpopedal spasm: flexion of the wrist and metacarpophalangeal joints, extension of the interphalangeal joints, and adduction of the thumb. She had an old, transverse post‐surgical scar on her neck. Ophthalmological evaluation revealed an intraocular lens implant in each eye. There was no pallor, icterus, clubbing, or lymphadenopathy. Her respiratory, cardiovascular, abdominal, and neurological examinations were unremarkable.

Laboratory investigations revealed the following findings: corrected serum total calcium, 1.25 mmol/L (normal range, 2.1–2.6); serum phosphorus, 2.19 mmol/L (normal range, 0.8–1.54); intact parathyroid hormone (IPTH), 5.2 ng/L (normal range, 7.5–53.5); 25‐hydroxyvitamin D, 112.07 nmol/L (normal range, 74.88–249.6); magnesium, 0.53 mmol/L (normal range, 0.69–1.02); serum albumin, 39 g/L (normal range, 38–49); alkaline phosphatase (ALP), 221 U/L (normal range, 90–306); free T3, 3.05 pmol/L (normal range, 4.26–8.1); free T4, 28.3 pmol/L (normal range, 10.2–28.2); thyroid‐stimulating hormone (TSH), 0.31 mU/L (normal range, 0.46–4.68); serum sodium, 138 meq/L (normal range, 135–145); and potassium, 5.1 meq/L (normal range 3.5–5). Her ECG showed normal sinus rhythm with no QT interval prolongation.

**FIGURE 1:**
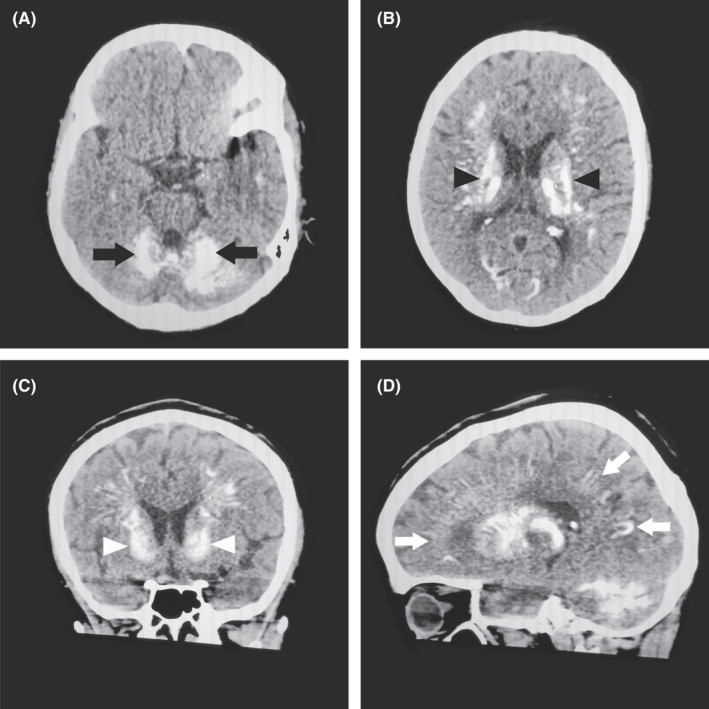
Plain computed tomography of the brain. Bilateral calcifications are seen in cerebellum (A), black arrows; basal ganglia (B) black arrowheads; thalami, periventricular region (C), white arrowheads; and subcortical fronto‐pareito‐occipital region (D), white arrows

Computed tomography of the brain revealed extensive bilateral calcifications involving basal ganglia, thalami, periventricular region, subcortical fronto‐parieto‐occipital region, and cerebellum (Figure 1).

The patient was treated with intravenous calcium gluconate, calcitriol, and magnesium. Serum calcium and magnesium levels were checked daily. After four days of intravenous calcium treatment, total calcium rose to 2.1 mmol/L. Her spasms subsided, and seizure did not recur. On the fifth day, intravenous calcium was replaced by oral calcium. She was discharged on 1500 mg of oral calcium and 1 mcg of oral calcitriol daily.

The patient was explained about the appropriate supplements and advised to follow‐up every three months. She followed up on the third month of discharge. She had strictly adhered to the treatment regimen and had not experienced seizure or spasm again; her total calcium level was 2.15 mmol/L (normal range, 2.1–2.6).

## 3 DISCUSSION

Hypoparathyroidism is the commonest postoperative complication after thyroid surgery and is characterized by hypocalcemia, elevated serum phosphorus levels, and low or inappropriately normal plasma levels of parathyroid hormone.^1^, ^4^ Postoperative permanent hypoparathyroidism is characterized by the persistence of hypocalcemia beyond six months of surgery.^1^, ^6^ Its incidence depends on the extent of thyroidectomy and lymph node dissection.^1^, ^2^, ^3^ Similarly, it occurs more frequently after surgeries done for thyroid cancer than those done for benign diseases.^4^

Hypocalcemia presents classically with muscle twitching, spasms, tingling, and numbness. Carpopedal spasm is characteristic and, in severe cases, can progress to tetany, seizures, and cardiac dysrhythmias. Signs of neuromuscular excitability can be observed on provocation tests: Chvostek's sign and Trousseau's sign.^7^ Hypocalcemia associated with chronic hypoparathyroidism exhibits several unique features such as basal ganglia calcifications, neuropsychiatric symptoms, and cataracts.^7^ While the exact mechanism behind cerebral calcification in hypoparathyroidism is still not clear, it does occur less frequently in postoperative hypoparathyroidism compared with idiopathic hypoparathyroidism.^8^ Patients with postoperative hypoparathyroidism tend to be supplemented with calcium and vitamin D earlier than those with idiopathic hypoparathyroidism. The resulting comparatively shorter duration of hypocalcemia in postoperative hypoparathyroidism is presumed to be the reason behind the fewer incidence of cerebral calcification.^8^ The calcifications typically involve caudate nucleus, putamen, globus pallidus, thalamus, and dentate nucleus; calcifications beyond these areas are rare.^5^, ^9^ Our patient had extensive bilateral intracranial calcifications, which involved the basal ganglia, thalamus, periventricular region, subcortical fronto‐parieto‐occipital region, and cerebellum. Some patients with basal ganglia calcifications develop Parkinsonism, while others, like our patient, do not.^5^, ^9^, ^10^ Posterior subcapsular cataract is common in patients with longstanding hypocalcemia.^6^ Our patient had also undergone bilateral cataract surgery eight years ago, but the type of cataract could not be ascertained as the surgical notes and documentation of the type of cataract were not available.

Generalized tonic‐clonic seizure is the most common type of seizure to occur in both idiopathic and postoperative hypoparathyroidisms.^11^ Modi et al^11^ found that idiopathic hypoparathyroidism patients who had seizure also had a higher frequency of basal ganglia calcification than those who did not have seizure, suggesting an association between them. They also found that supplementing calcium and vitamin D provided a better control of seizures than giving antiepileptic drug alone. That was evident when some patients on antiepileptic drug alone, with poor control of seizures, improved after getting diagnosed with idiopathic hypoparathyroidism, and supplemented with calcium and vitamin D. Additionally, calcium and vitamin D supplementation with the maintenance of serum calcium level at 1.8 mmol/L was found to prevent seizure recurrence irrespective of whether the patients were taking antiepileptic drugs or not. Our patient presented with a single episode of generalized tonic‐clonic seizure. She was treated with intravenous calcium gluconate, calcitriol, and magnesium and did not have seizure recurrence during her hospital stay as the laboratory parameters corrected to normal levels. She was not given antiepileptic drugs at the time of discharge but was advised for close monitoring.

Parathyroid hormone increases renal tubular calcium reabsorption and stimulates osteoclastic bone resorption to raise serum calcium levels.^7^ It also enhances the synthesis of 1,25‐dihydroxyvitamin D in the kidneys, which then absorbs calcium from the intestine.^7^ Inadequacy of parathyroid hormone impairs these functions and results in hypocalcemia. Patients with permanent hypoparathyroidism require life‐long calcium supplementation.^6^ The goal is to maintain serum calcium in the low normal range and check serum calcium every three to six months or when the medical regimen is changed.^6^ Additionally, hypoparathyroidism warrants supplementation of 1,25‐dihydroxyvitamin D for optimum maintenance of normal calcium levels. Commonly available vitamin D preparations contain ergocalciferol or cholecalciferol, both of which are ineffective in hypoparathyroidism for the aforementioned reasons.^7^ Our patient stopped checking her calcium levels one year ago, and then five months ago, she stopped taking calcium and vitamin D supplements as well because she felt well. Within a month of stopping the supplements, she experienced carpopedal spasm, which was empirically treated with brief supplementation of calcium and cholecalciferol without checking her serum calcium levels. Calcitriol was not prescribed, either. This, most likely, led to the severe hypocalcemia that she had on presentation.

Magnesium level in our patient was 0.53 mmol/L (normal range, 0.69–1.02). Mild hypomagnesemia stimulates the secretion of Parathyroid hormone (PTH), but, severe hypomagnesemia (below 0.5 mmol/L), paradoxically, reduces PTH secretion.^12^ This paradoxical effect of magnesium on PTH secretion is due to the effect of decreased intracellular magnesium on the Calcium‐sensing receptor‐associated G protein subunit.^12^ Since our patient had mildly decreased magnesium levels, the possibility of hypomagnesemia causing decreased PTH secretion is unlikely. The principal reason for infusing magnesium in our patient was to mitigate the increased risk of cardiac arrhythmia in the face of severely low calcium levels, rather than increasing PTH secretion from parathyroids.

## 4 CONCLUSION

Postoperative permanent hypoparathyroidism can lead to extensive bilateral intracranial calcifications involving basal ganglia, thalamus, cerebellum, and cerebral cortex. Severe hypocalcemia can occur even after several years of surgery; therefore, serum calcium levels of individuals with postoperative permanent hypoparathyroidism should be measured life‐long at regular intervals. Hypoparathyroidism should be considered in post‐thyroidectomy individuals who present with seizures or intracranial calcifications.

## CONFLICT OF INTEREST

The authors declare that they have no competing interests.

## AUTHOR CONTRIBUTIONS

PC and SK: wrote the initial draft of the manuscript. RO, NG, MS, and RB: reviewed the manuscript. PC and SK: edited the draft and reshaped it into this manuscript. All authors read and approved the final manuscript.

## ETHICAL APPROVAL

Need for ethics approval waived. Consent from the patient deemed to be enough.

## CONSENT

Written informed consent was obtained from the patient for publication of this case report and any accompanying images.

## Data Availability

The data that support the findings of this study are available from the corresponding author upon reasonable request.
